# Phylogenetic Analysis of *Fusarium solani* Associated with the Asian Longhorned Beetle, *Anoplophora glabripennis *

**DOI:** 10.3390/insects3010141

**Published:** 2012-02-10

**Authors:** Scott M. Geib, Erin D. Scully, Maria del Mar Jimenez-Gasco, John E. Carlson, Ming Tien, Kelli Hoover

**Affiliations:** 1 Tropical Crop and Commodity Protection Research Unit, USDA-ARS Pacific Basin Agricultural Research Center, 64 Nowelo Street, Hilo, HI 96720, USA; 2 Intercollege Program in Genetics at The Huck Institutes of the Life Sciences, The Pennsylvania State University, University Park, PA 16802, USA; E-Mail: eds14@psu.edu; 3 Department of Plant Pathology, The Pennsylvania State University, University Park, PA 16802, USA; E-Mail: jimenez-gasco@psu.edu; 4 School of Forest Resources, The Pennsylvania State University, University Park, PA 16802, USA; E-Mail: jec16@psu.edu; 5 Department of Bioenergy Science and Technology (World Class University), Chonnam National University, Buk-Gu, Gwangju 500-757, Korea; 6 Department of Biochemistry and Molecular Biology, The Pennsylvania State University, University Park, PA 16802, USA; E-Mail: mxt3@psu.edu; 7 Department of Entomology and Center for Chemical Ecology, The Pennsylvania State University, University Park, PA 16802, USA

**Keywords:** wood-feeding insect, fungal symbiosis, Nectria haematococca

## Abstract

Culture-independent analysis of the gut of a wood-boring insect, *Anoplophora glabripennis* (Coleoptera: Cerambycidae), revealed a consistent association between members of the fungal *Fusarium solani* species complex and the larval stage of both colony-derived and wild *A. glabripennis* populations. Using the translation elongation factor 1-alpha region for culture-independent phylogenetic and operational taxonomic unit (OTU)-based analyses, only two OTUs were detected, suggesting that genetic variance at this locus was low among *A. glabripennis*-associated isolates. To better survey the genetic variation of *F. solani* associated with *A. glabripennis,* and establish its phylogenetic relationship with other members of the *F. solani* species complex, single spore isolates were created from different populations and multi-locus phylogenetic analysis was performed using a combination of the translation elongation factor alpha-1, internal transcribed spacer, and large subunit rDNA regions. These analyses revealed that colony-derived larvae reared in three different tree species or on artificial diet, as well as larvae from wild populations collected from three additional tree species in New York City and from a single tree species in Worcester, MA, consistently harbored *F. solani* within their guts. While there is some genetic variation in the *F. solani* carried between populations, within-population variation is low. We speculate that *F. solani* is able to fill a broad niche in the *A. glabripennis* gut, providing it with fungal lignocellulases to allow the larvae to grow and develop on woody tissue. However, it is likely that many *F. solani* genotypes could potentially fill this niche, so the relationship may not be limited to a single member of the *F. solani* species complex. While little is known about the role of filamentous fungi and their symbiotic associations with insects, this report suggests that larval *A. glabripennis* has developed an intimate relationship with *F. solani* that is not limited by geographic location or host tree.

## 1. Introduction

The Asian longhorned beetle (*Anoplophora glabripennis*) is an invasive, wood-boring insect with a relatively broad host range that now includes over 21 deciduous tree species [1]. *A. glabripennis* was first detected in the United States in 1996 and, since its arrival, has caused millions of dollars in damage to urban streetscapes in several northeastern and midwestern states. It also poses a threat to the maple syrup industry and forest ecosystems [2,3]. Most cerambycids are constrained to feeding in stressed, dying, or dead trees and are reported to digest cellulose by ingesting enzymes produced by wood-degrading fungi that colonize infected wood [4,5]. In contrast, other Lamiinae, including larval *A. glabripennis,* feed and grow in the inner wood of a variety of healthy hardwood tree species where woody, intractable components, including lignin and cellulose, have not been pre-digested by wood-degrading fungi and, instead, are internally digested [6,7,8,9]. 

Although many cerambycids have the endogenous capacity to produce endoglucanases and glycoside hydrolases capable of disrupting random β-1,4 linkages in cellulose chains and hydrolyzing β-1,4 linkages in cellobiose disaccharides, respectively [10,11,12], insects do not produce exoglucanases, which processively cleave cellobiose and other cello-oligosaccharides from reducing and non-reducing ends of cellulose polymers [13] These exoglucanases are essential to efficiently liberate glucose from carbohydrate polymers; while they are produced by a number of cellulose-degrading bacteria and fungi, no definitive insect- or animal- derived exo-type glucanases have been conclusively identified [10,12]. In addition, cellulose, hemicellulose, and small amounts of protein are often cross-linked to lignin in hardwood tree species. Thus, circumventing the lignin barrier is paramount for accessing hexose and pentose sugars present in cellulose and hemicellulose polymers and for protein acquisition. Although lignin degradation has been well-documented in the *A. glabripennis* gut [14], no known insect-derived enzymes are capable of catalyzing the oxidative reactions responsible for large-scale lignin depolymerization in this system and, in fact, only fungi are known to produce peroxidases capable of catalyzing the types of lignin-directed reactions observed in larval *A. glabripennis*. Furthermore, *A. glabripennis* larvae face a number of other obstacles as they feed in the sapwood and heartwood of healthy host trees, including low protein/nitrogen availability, low abundance of essential dietary nutrients (e.g. sterols, fatty acids, and vitamins), and toxic secondary metabolites produced by the host tree [15]. 

Thus, it is likely that microbial species are important in aiding in wood degradation and nutrient acquisition within *A. glabripennis*. In the wood-feeding beetle species examined to date that harbored bacteria, a broader diversity of gut microbes was associated with broader tree host range. Raffa and colleagues [16] found a broad diversity of bacteria in the gut of *A. glabripennis* larvae from willow trees in China, while the linden borer (*Saperda vestita*), a cerambycid with a more restricted host range, contained only a small subset of these same bacteria [17]. Follow-up studies examining the bacterial community composition from colony derived and wild *A. glabripennis* populations collected from several suitable host trees revealed that the community was consistently dominated by proteobacteria and actinobacteria, including many taxa capable of degrading small aromatic lignin metabolites, cellulose, and xylan, but displayed a considerable degree of plasticity at lower taxonomic levels. In addition, larvae fed on a cellulose-based artificial diet, containing bacteriostatic agents displayed a substantially lower community diversity coupled with reduced endo- exo- and β-glucosidase activities in comparison to tree-fed larvae, suggesting that bacteria may be contributing to cellulase enzyme production in the gut. In concert, a number of carboxymethylcellulose degrading bacteria were successfully cultured from insects feeding on suitable host trees, including bacteria belonging to the families Brevobacteriaceae, Burkholderiaceae, Micrococcaceae, Staphylococcaceae, and Streptomycetaceae [18]. Several of these bacterial taxa are found in association with larval *A. glabripennis* throughout its life cycle, including bacteria from the families Bacilliaceae and Xanthomonadaceae , suggesting that they may be vertically transmitted during oviposition [19]. 

In several beetle species studied to date, a number of fungal species have been discovered in association with the insect’s gut, including yeast endosymbionts [20,21,22,23]. The most thoroughly-characterized are yeasts in the basidiocarp-dwelling beetles (mushroom-eating beetles), in which about 300 species of yeasts have been discovered in 25 families [21,24]. Some of these yeasts produce xylanases and other symbiotic yeasts are speculated to play roles in nitrogen metabolism, detoxification, and nutrient synthesis [20,21,25,26,27,28,29,30]. Some cerambycids harbor intracellular yeasts in mycetocytes, specialized cells in the gastric caecae located at the junction between the foregut and midgut [25]. These yeasts are maternally transmitted by inoculation of the egg surface; newly hatched larvae ingest the egg membrane, acquiring yeasts in the process [25]. Thus far, all yeasts isolated from cerambycids are in the genus *Candida* [31,32]. In addition to yeast-like endosymbionts, some beetles are occasionally found in non-pathogenic interactions with members of the *Fusarium solani* species complex, a group of metabolically diverse fungi dominated by notorious plant and animal pathogens [33]. The most well-studied relationship to date is the relationship between *F. solani* and ambrosia beetles: in these symbioses, ambrosia beetles harbor fungal spores in mycangia, inoculating the fungus into woody tissue, where it synthesizes sterols required for pheromone production and aids in cellulose digestion [34]. While *Fusarium moniliforme* and *Fusarium roseum var. graminearum* found in association with *Tribolium confusum* often have profound, positive impacts on fecundity and fitness, the nature of the relationship is poorly understood and their precise contributions to nutrient acquisition, digestive processes, and insect physiology have yet to be characterized [33]. A considerable amount of research has been devoted to characterizing the fungal communities of other cerambycids, yet very little is known about the fungal communities of the Lamiinae, including *A. glabripennis*. Interestingly, the Lamiinae were initially thought to lack fungal endosymbionts or to possess mycetocytes until culture-based molecular techniques recently detected the presence of several different ascomycete yeast species found in association with beetle guts (Lamiinae) collected from neotropical regions [32]. In previous studies in our lab, *F. solani* was detected in association with larvae collected from wild populations in New York City [14], but the consistency of this relationship, and the persistence of this relationship in other beetle populations, was not assessed. The goal of this research was to identify and phylogenetically characterize fungal isolates collected from the guts of larval *A. glabripennis* from different geographic locations and host tree species.

## 2. Materials and Methods

### 2.1. Rearing Colony Derived *A. glabripennis* on Different Host Trees

Larvae were reared in nursery lines of one of three tree species: sugar maple (*Acer saccharum*), pin oak (*Quercus palustris*), and callery pear (*Pyrus calleryana*), as described previously [18]. Trees were planted in 20-gallon nursery containers filled with Fafard 52 pine bark medium (Fafard Inc., Agawam, MA, USA), and grown at an outdoor pot-in-pot nursery at the Pennsylvania State University, University Park campus until they were 4–5 years old. Several weeks prior to use in experiments, trees were moved into a quarantine greenhouse to allow for acclimation to greenhouse conditions. Experiments had to be conducted under quarantine conditions due to the quarantine regulations of *A. glabripennis.* Three trees of each species were placed in large (ca. 3 m high, 3 m long, 2 m wide) walk-in insect cages, each cage containing only one tree species, and maintained as described previously [8]. Adult *A. glabripennis* were obtained from a quarantine research colony of mixed ancestry [35]. The research colony is maintained on a cellulose-based artificial diet [36] using Norway maple (*Acer platanoides*) bolts for adult feeding and oviposition. For this experiment, three mated pairs of adults per tree species (n = 9 pairs) were maturation fed on twigs from either sugar maple or pin oak for 3-5 days. After maturation, mating pairs were released into a cage containing potted trees of the same tree species from which they maturation fed, and were allowed to oviposit into these trees for 2 weeks. At this point, adults were removed from the cages. The trees were then held in the greenhouse for 90 days to permit larval establishment. After 90 days, each tree was dissected and living larvae were collected for gut community analysis. 

Callery pear was not used for maturation feeding or oviposition because *A. glabripennis* do not survive or produce eggs when fed on twigs of this tree species and eggs laid into callery pear do not survive [35]. Because *A. glabripennis* are reluctant to feed on, and do not grow well in callery pear [35], a portion of the larvae extracted from sugar maple were then manually inserted into callery pear and allowed to feed for 2 weeks as described previously [37] to examine the effect of this resistant host on the gut community. Callery pear trees were dissected 2 weeks after larval insertion and apparently healthy, feeding larvae were collected for gut community analysis. Larvae that fed on a cellulose-based artificial diet [36] were also collected for gut community analysis from the quarantine lab colony, of approximately the same age as the tree-reared larvae. While the artificial diet does not contain any specific antibiotics, it does include components such as sodium propionate, sorbic acid, and p-hydroxybenzoic acid methyl ester, which have fungal and microbial inhibitory properties [38]. Samples from these greenhouse trees and quarantine colony will be referred to as “PSU colony-derived” from this point on. 

### 2.2. Collection of Larval *A. glabripennis* and Fungal Cultures from Introduced Wild Populations

To compare fungal community composition of PSU colony-derived larvae to field-derived insects, *A. glabripennis* were collected from field populations located in Brooklyn, New York, United States (referred to as NYC-derived), and Worcester, Massachusetts, United States (referred to as MA-derived) in conjunction with eradication efforts by USDA-APHIS-PPQ. Infested trees were located based on the presence of exit holes and dieback. Four trees were cut for this study from New York City, including two silver maples (*Acer saccharinum*), one sycamore maple (*Acer pseudoplatanus*), and one horse-chestnut (*Aesculus hippocastanum*). From Worcester, Massachusetts, infested material was collected from two silver maple trees.

Trees were cut into segments on site and transferred to the lab where they were dissected to remove larvae. Larvae were immediately frozen after removal from tree segments and were stored at −80 °C until use. 

### 2.3. *Anoplophora Glabripennis* Larval Gut Dissection and DNA Extraction

Larval dissections were performed using sterile dissection tools in a laminar flow hood to maintain sterility. Larvae removed from PSU colony-derived trees were immediately chilled and dissected within 1 hour of removal from trees. NYC- and MA-derived larvae were kept frozen until immediately before dissection, and were then maintained on ice. Larvae were surface sterilized in 70% ethanol for 1 min and rinsed in sterile water before dissection. Whole guts were dissected by cutting the cuticle open laterally, ligating the gut at the anterior midgut and posterior hindgut, and carefully transferring the entire gut into a sterile microcentrifuge tube. For the PSU-colony and NYC-derived samples, guts from 10 larvae feeding on a single tree were pooled into a single microcentrifuge tube for DNA extraction. Then, total DNA was extracted using the FastDNA® SPIN for Soil Kit (MP Biomedicals), using the FastPrep® Instrument for tissue homogenization, following the manufacturer's protocol. This kit was used due to the complexity of the *A. glabripennis* gut contents (containing wood, bacteria, fungi) to ensure complete DNA extraction from all organisms and removal of wood polysaccharides and secondary metabolites that can co-extract with DNA. A control DNA extraction was also performed using the sterile water rinsate to confirm external sterilization of larvae. DNA concentration was determined by absorbance at 260 nm and extracts were stored at −20 °C until use. A subset of gut tissue from PSU-colony and NYC-colony derived insects and all MA-derived insects were used for fungal culturing.

### 2.4. Culture Independent Fungal Community Analysis

Initially, the internal transcribed spacer region (ITS1-ITS4) was amplified and sequenced from total gut DNA extractions from each treatment, a commonly used genomic region for fungal identification [39]. After preliminary experiments from which only *Fusarium* strains were identified, the translation elongation factor-1 alpha (TEF1-α) region was used instead for culture independent analysis, due to the potential presence of non-orthologous copies of the ITS region in some *Fusarium* that can lead to incorrect identification [40,41]. Eight culture independent libraries were created, one for each of the following DNA samples: PSU colony-derived sugar maple, PSU colony-derived pin oak, PSU colony-derived callery pear, PSU colony-derived artificial diet, NYC-derived silver maple tree 1, NYC-derived silver maple tree 2, NYC-derived horse-chestnut, and NYC-derived sycamore maple. Fungal-specific primers for translation elongation factor 1 (ef1, 5’-ATG GGT AAG GA(A/G) GAC AAG AC-3’) and translation elongation factor 2 (ef2, 5’-GGA (G/A)GT ACC AGT (G/C)AT CAT GTT-3’) were used to amplify the TEF1-α region, which can amplify this region from a broad range of filamentous ascomycetes [42,43]. PCR reactions were performed in 25 μL volumes with the following components: 5 μL of 5× GoTaq green reaction buffer, 0.5 μL GoTaq DNA polymerase (1.25 U, Promega, Madison, WI), 1 μL 10 μM dNTP mix, 2 μL of 10 μM forward primer (ef1), 2 μL of 10 μM reverse primer (ef2), and 20 ng of template DNA. PCR conditions were 95 °C denaturation for 3 min, 25 cycles of 95 °C for 30 sec, 53 °C for 45 sec, 72 °C for 1:30 min, with a final extension at 72 °C for 5 min. PCR reactions were also performed on control DNA to ensure that there was no contaminating DNA during extraction. Agarose gel electrophoresis verified amplification of target DNA and confirmed that no fungal products were amplified from sterile rinsate controls. For each gut pool, 2 μL of the PCR product was ligated into the pCR® 2.1 TOPO vector (Invitrogen, Carlsbad, CA) following the manufacturer’s protocol. The vector was then transformed into chemically competent *E. coli* cells (TOP10, Invitrogen, Carlsbad, CA) by heat-shock and clone libraries were created. 

Insert DNA from clones was amplified from the M13 priming site of the vector using direct PCR. Twenty-five μL PCR reactions were set up in 96-well format with the following components: 5 μL of 5× GoTaq green reaction buffer, 0.5 μL GoTaq DNA polymerase (1.25 U, Promega, Madison, WI), 1 μL 10 μM dNTP mix, 2 μL of 10 μM forward primer (M13 Universal), and 2 μL of 10 μM reverse primer (M13 Rev). For each of the 8 clone libraries, 48 colonies were picked from the clone library using a sterile pipette tip and immersed into the PCR mix to add bacterial cells to the PCR reaction. The PCR program included an initial 95 °C denaturation for 10 min to rupture bacterial cells, followed by 30 cycles of 95 °C for 30 sec, 55 °C for 1:00 min, 72 °C for 1:30 min, with a final extension at 72 °C for 5 min. Amplification was confirmed by gel electrophoresis; for successful amplifications, 4 μL of the PCR product was purified for sequencing by adding 0.8 μL of ExoSAP-IT (USB Corporation, Cleveland, OH) and incubating the sample at 37 °C for 15 min, followed by 80 °C for 30 min. Two μL of this reaction were then used to sequence from the forward direction from the M13 Universal priming site and 2 μL for the reverse from the M13 Rev site using BigDye chemistry at the Penn State Genomics Core Facility. 

### 2.5. Aerobic Culturing of Gut Fungus on Restrictive Media

A subset of the PSU-colony derived larvae from sugar maple trees, a NYC-derived larva from sycamore maple, and all MA-derived larvae were used to aerobically culture fungi present in the gut. This enabled the establishment of individual cultured isolates, and thus, a more-in-depth phylogenetic analysis encompassing multiple loci could be performed, overcoming a major limitation of culture-independent approaches. Larval dissections were performed in a laminar flow hood. For PSU-colony derived, NYC-derived, and MA-derived insects, five guts from insects from a given host tree were pooled into a single microcentrifuge tube containing 500 μL of sterile PBS solution (0.01M, 0.138M NaCl, 0.0027 M KCl, pH 7.4). For the PSU-colony, three different sugar maple trees produced enough larvae to create three independent cultures. For MA-derived larvae, two silver maple trees produced sufficient larvae to produce cultures. For NYC-derived larvae, only a single sycamore maple produced a sufficient number of larvae for subculturing. A control tube containing only PBS was also used for inoculation to ensure there was no contamination during dissection and plating. 

Tissues were homogenized using a disposable micropestle and vortexed at a medium speed for 30 seconds. Serial dilutions of each pooled homogenate were performed in PBS (1:10, 1:100, 1:1000, and 1:10000). One hundred μL of each dilution were plated in triplicate onto amended CMC agar plates [17] (5 g carboxymethyl cellulose, 10 g tryptic soy broth, 0.03 g malt extract, and 12 g agar for 1 L, pH 7.0) treated with tetracycline and incubated at 28 °C for 2–4 days. Single spores from several representative fungal colonies from each pool of guts were sub-cultured on tetracycline amended potato dextrose agar (BD, Franklin Lakes, New Jersey, USA) to generate monoconidial cultures. The monoconidial cultures were subcultured onto potato dextrose “reverse agar” (potato dextrose broth with 30% BASF pluronic polyol F-127), and the fungal cultures were easily collected from this media by refrigerating the plates at 4 °C to re-liquefy media, and then spinning down the mycelia at 4 ºC at 12,000 × g for 10 min in a 50 mL centrifuge tube. At this point, the supernatant was removed and the mycelia were washed with TE buffer to remove remaining media. 

DNA extraction was performed by grinding the collected mycelia under liquid nitrogen, followed by extraction in 5 volumes of extraction buffer (50 mM Tris-HCl pH 8.0, 50 mM EDTA, 3% SDS, 0.1 mg/mL protease K, and 1% β-mercaptoethanol) at 65 °C for 1 hour. Phenol:chloroform extraction was then performed with two rounds of extraction in phenol:chloroform:isoamyl alcohol (25:24:1), followed by 1 round of chloroform:isoamyl alcohol (24:1). DNA was precipitated from the upper phase by addition of 0.5 volume 7 M ammonium acetate and 2 volumes of 95% ethanol and incubated for 1 hour at −20 °C. DNA was pelleted and resuspended in TE buffer and quantified by measuring absorbance at 260 nm. The number of cultures analyzed for each sample is displayed in Table 1.

### 2.6. Multi-Locus Sequencing from Cultured Fungal DNA Extraction

PCR was performed on each single-spore isolate DNA extract to amplify the TEF1-α region as described above [44]. In addition, PCR was performed on the ITS and NL regions of the rRNA [39]. The ITS region was amplified by using PCR primers ITS5 (5'-GGAAGTAAAAGTCGTAACAAGG-3') and ITS4 (5'-GGTCCGTGTTTCAAGACGG-3'), which encompasses the end of the 18S rDNA locus, the entire ITS1-5.8S rDNA-ITS2 region, and the beginning portion of the 28S rDNA locus. The LSU 28s rDNA region was amplified with primers NL1 (5'-GCATATCAATAAGCGGAGGA-3') and NL4 (5'-GGTCCGTGTTTCAAGACGG-3'). PCR reactions were purified for sequencing as described above and sequenced in both directions using 2 µL of 1 µM forward or reverse primer to prime the sequencing reaction. All sequencing was performed using BigDye chemistry at the Penn State Genomic Core Facility.

**Table 1:** Number of monoconidial gut-derived fungal cultures from each host tree.

		Tree Replicate Number			
	Host tree	1	2	3	Total
PSU-derived	Sugar Maple	7	6	3	16
NYC-derived	Sycamore Maple	1	-	-	1
MA-derived	Silver Maple	4	1	-	5
	Total	12	7	3	22

### 2.7. Sequence Editing, Alignment and Operational Taxonomic Unit (OTU) Analysis of TEF1-α

Alignment of forward and reverse TEF1-α sequences for each sample from both cultured and culture independent samples was performed using Sequencher 4.8 (Gene Codes Corporation, Ann Arbor, MI). After alignment, the cloning vector sequence was removed and a single consensus sequence for each clone was created by manually discriminating conflicting base calls between the forward and reverse reads. All edited sequences obtained from culture-independent analyses and cultured isolates were submitted to Genbank under accession numbers JQO25721 – JQ025987 and JN9803020 – JN9803041, respectively. Using the TEF1-α consensus sequences from all clone libraries and all cultured samples, a ClustalW alignment was generated using MEGA4 [45] and then manually edited to improve accuracy of alignment. This dataset was then used to perform operational taxonomic unit (OTU) analysis using the mothur software package [46]. First, a Jukes-Cantor distance matrix was produced using the DNAdist program in the Phylip package using default parameters. This distance matrix was then subjected to OTU cluster analysis with mothur, using the furthest neighbor algorithm and a cutoff of 0.97 [46]. This analysis binned all sequences into two OTUs. BLASTn comparison of a representative sequence from each OTU to the nt and FusariumID databases confirmed that both OTUs contained sequences within the *F. solani* species complex [42]. 

### 2.8. Single- and Multi-locus Phylogenetic Analysis

For phylogenetic reconstruction and placement of cultures within the *F. solani* species complex, we utilized the dataset of O’Donnell [44], which reconstructed the phylogeny of the *F. solani* complex using the TEF1-α, ITS, and LSU regions and included a broad range of *F. solani* isolates from a variety of geographically distinct locations, mating populations, and subspecies. The nexus file used in this analysis was downloaded from TreeBASE [47]. ITS, TEF1-α, and LSU sequences obtained from *A. glabripennis*-derived cultures were concatenated together and manually aligned to the O'Donnell dataset. This alignment was partitioned into individual loci by position using PAUP Beta 4.0 (Windows) and this partitioned dataset was used for all single locus and multilocus phylogenetic reconstructions [48]. ITS and LSU sequences obtained from cultured isolates were submitted to Genbank under the accession numbers JN982998–JN983019 and JN982967–JN982997, respectively.

Using the sequences obtained from culture-independent analysis of the TEF1-α locus, a single representative sequence was randomly selected from each OTU for inclusion into the TEF1-α phylogenetic analysis (OTU 1: F01_HEREF (from NYC-derived horse chestnut) and OTU 2: B11DEF_34 (from PSU colony-derived artificial diet). These OTU sequences were manually aligned to the TEF1-α locus (bases 585 to 1290 of the multilocus alignment) and the TEF1-α alignment was extracted and imported into jModelTest (version 0.1.0) to select best fit models of nucleotide substitution optimized for maximum likelihood tree topology using Akaike Information Criteria (AIC) [49,50]. Maximum likelihood trees were estimated using the TPM2uf + G evolutionary model with GARLI 2.0 (Genetic Algorithm for Rapid Likelihood Inference) [51]. Evolution was simulated for 500,000 generations or until likelihood scores reached convergence; nonparametric bootstrap analysis was conducted to generate support for branching topology (n = 500 pseudoreplicates). Bootstrap consensus trees were rooted with *Fusarium staphyleae* and compiled using SumTrees version 3.3.1 [52]. Nodes with branch lengths <1E-08 were collapsed and bootstrap values for nodes with support >50 are reported. 

Phylogenetic analysis using ITS and LSU loci was also conducted using the O’Donnell dataset and ITS and LSU sequences obtained from cultured *A. glabripennis-*derived *F. solani* isolates; however, since ITS and LSU sequences were not available from culture-independent analysis, representative sequences from OTUs could not be included. Maximum likelihood trees were simulated using TIM + I + G for ITS and TIMef + I for LSU with GARLI [31,32,33]. Evolutionary simulation, nonparametric bootstrap analysis, and compilation of bootstrap consensus trees were performed in the same manner as described above. 

Single locus phylogenetic reconstructions were of limited use in resolving the relationship of *A. glabripennis*-derived isolates to the *Fusarium solani* species complex due to a large number of unresolved nodes and nodes with low bootstrap values, particularly using the ITS and LSU loci (data not shown). In an attempt to improve bootstrap support values and resolve multifurcating nodes, a multilocus approach was used to construct maximum likelihood phylogenetic trees. Maximum likelihood trees were computed using GARLI [33]. Each locus was treated as a separate partition and allowed to evolve independently according to its optimal model of nucleotide substitution determined by jModelTest (ITS: TIM + I + G; EF: TPM2uf + G; LSU: TIMef + 1) [31,32]. Evolution was simulated for 500,000 generations or until likelihood scores reached convergence and nonparametric bootstrap analysis was performed to generate support for branching patterns (n = 500 pseudoreplicates). Bootstrap consensus trees were rooted with *F. staphyleae* and were compiled as described above. 

## 3. Results and Discussion

### 3.1. Culture Independent Fungal Community Analysis

In total, 277 TEF1-α clones associated with *A. glabripennis* were sequenced and analyzed. From this total, 153 were derived from insects from the Penn State research colony, with 39 clones from insects reared on sugar maple, 33 clones from insects reared on pin oak, 46 clones from insects reared on callery pear, and 35 clones from insects reared on cellulose based artificial diet (Table 2). The remaining 124 clones were from insects from field populations in New York City, with 30 clones from insects collected from silver maple tree 1, 32 clones from insects collected from silver maple tree 2, 29 clones from insects collected from a horse-chestnut tree, and 33 clones from a sycamore maple tree. Based on OTU analysis, using the furthest neighbor algorithm, all TEF1-α sequences derived from culture-independent methods were categorized into two OTUs; specifically, 268 TEF1-α sequences were placed into OTU1, while only nine TEF1-α sequences were placed into OTU2. In addition, while OTU2 was found in association with PSU-derived larvae fed on pin oak or artificial diet, it was not found in association with any *A. glabripennis* larvae derived from the NYC population. In contrast, OTU1 was highly abundant in both PSU- and NYC-derived populations and was detected in larvae sampled from all host tree species. 

**Table 2:** Distribution of culture independent TEF1-α clones from PSU colony- and NYC-derived larvae into Operational Taxonomic Units (OTUs). All 277 sequences clustered into two distinct OTUs. OTU1 was the most abundant OTU as it was detected in all NYC- and all PSU-derived samples. OTU2 was not detected in any of the NYC samples, but was detected in a subset of the PSU samples.

Culture Independent	Host tree	OTU Identification Number		
		1	2	Total
PSU-derived	Sugar Maple	39		39
	Pin Oak	27	6	33
	Callery Pear	46		46
	Artificial Diet	32	3	35
NYC-derived	Silver Maple (tree 1)	30		30
	Silver Maple (tree 2)	32		32
	Sycamore Maple	33		33
	Horse-Chestnut	29		29
Total		268	9	277

### 3.2. Culture-Dependent Fungal Analysis

A total of 22 monoconidial fungal cultures was obtained and analyzed across three loci. Based on analysis of the TEF1-α locus sequences, all of the cultured isolates were classified into the same two OTUs that were described in the culture-independent analysis (Table 3); specifically, nine PSU-derived cultures and one NYC-derived culture were categorized into OTU1, while eight PSU-derived and six MA-derived cultures were categorized into OTU2. In NYC- and MA-derived larvae, only representatives from OTU1 and OTU2 were cultured respectively; however, although OTU2 had not been detected previously in PSU-derived larvae reared on sugar maple, cultures from both OTUs were isolated from PSU-larvae. The abundance of OTU1 and OTU2 among *Fusarium* cultures generated from PSU larvae was roughly equivalent. 

**Figure 1:**
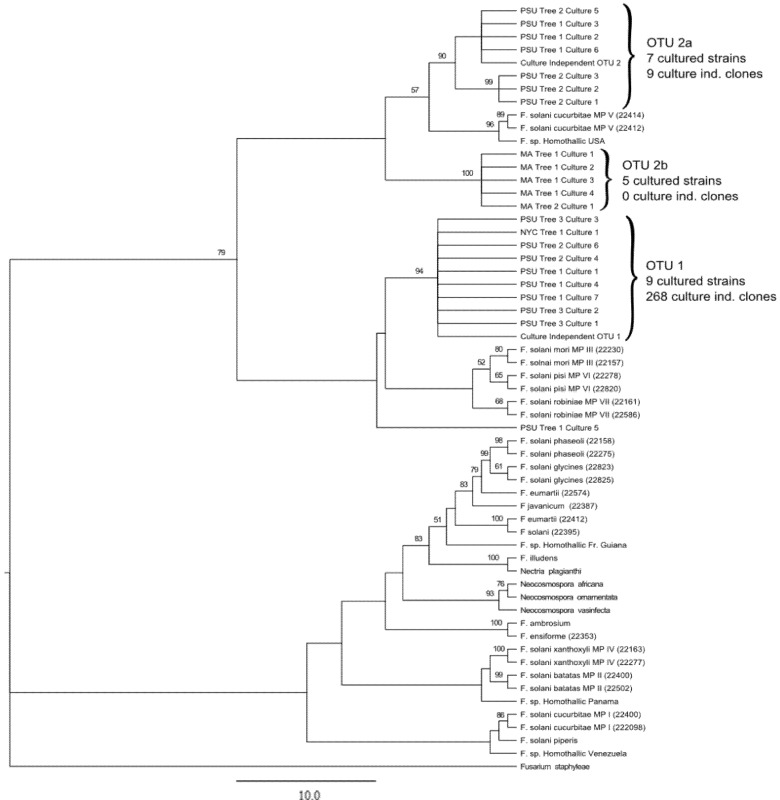
Phylogenetic analysis of ALB-derived and O’Donnell *Fusarium solani* isolates using the translation elongation factor 1-alpha locus. A rooted maximum likelihood tree was generated using TEF1-α sequences from all cultured representatives and a single representative from each OTU detected through culture-independent methods (n = 500 bootstrap replicates). These sequences were placed within the *F. solani* species complex, utilizing the dataset of O’Donnell [44]. Mating populations (abbreviated MP) are listed based off of the O’Donnell dataset. Nodes with bootstrap support values > 50 are reported, ML distance scale bar = 10 changes. ALB-derived isolates formed 3 strongly supported clusters, denoted as OTU1, OTU2A, and OTU2B. A single NYC isolate and all culture-independent sequences generated from this population were grouped into OTU1, while all MA-derived isolates were classified into cluster 2b. PSU-derived isolates can be observed in both OTU1 and OTU2a.

Phylogenetic analysis of both culture independent and cultured strains using the TEF1-α locus produced congruent results with OTU-based analysis (Figure 1). Broadly speaking, all *A. glabripennis* -derived sequences were resolved into two distinct clades, which corresponded well with the OTU classifications. Specifically, clade 1 contains the same nine cultured representatives that were categorized as OTU1, including a single isolate from NYC-derived larvae, nine PSU-derived isolates, and a representative culture-independent sequence from OTU1. Of particular interest is the low degree of sequence variation among isolates that fall into this clade, which is indicated by low branch lengths and unresolved nodes. While most sequences designated as OTU1 in the OTU-based cluster analysis form a monophyletic group distinct from the O'Donnell isolates, a single outlier (PSU Tree 5 Culture 1) that was categorized as OTU1 was present. Although no O'Donnell sequences were present in clade 1 (not including the outlier “PSU Tree 5 Culture 1”), the nearest group contains *F. solani* isolates from mating populations (listed as MP in figures based on O’Donnell analysis) III, VI, and VII (ff. spp. *mori, pisi* and *robiniae*, respectively). 

**Table 3:** Placement of cultured strains into Operational Taxonomic Units using TEF1-α locus. All PSU-, NYC-, and MA-derived cultured isolates were classified into the same two OTUs that were detected in the culture-independent analysis.

Cultured	Host tree	OTU Identification Number		
		1	2	Total
PSU	Sugar Maple	9	8	17
NYC	Horse chestnut	1	0	1
MA	Silver Maple	0	6	6
Total		10	14	24

Although all sequences from OTU2 formed a clade distinct from OTU1, there is a significantly higher degree of sequence heterogeneity within this group relative to OTU1 and this group can be further divided into two sub-clades (clades 2a and 2b). Clade 2a contains only isolates derived from PSU sugar maple-reared larvae and has a relatively high degree of within-group sequence heterogeneity, while clade 2b contains only isolates derived from MA populations and has a low degree of within-group sequence heterogeneity, indicated by unresolved nodes. Both clade 2a and 2b fall in a well-supported and distinct clade with the nearest O’Donnell isolate from *F. solani* f. sp. *cucurbitae* mating population V. Single locus analysis using the ITS and LSU rRNA regions from the cultured fungal isolates showed similar branch topologies as the TEF1-α derived tree but with much lower bootstrap values and many unresolved nodes (data not shown). These results are similar to results from these loci in the original analysis performed by O’Donnell [44]. 

**Figure 2:**
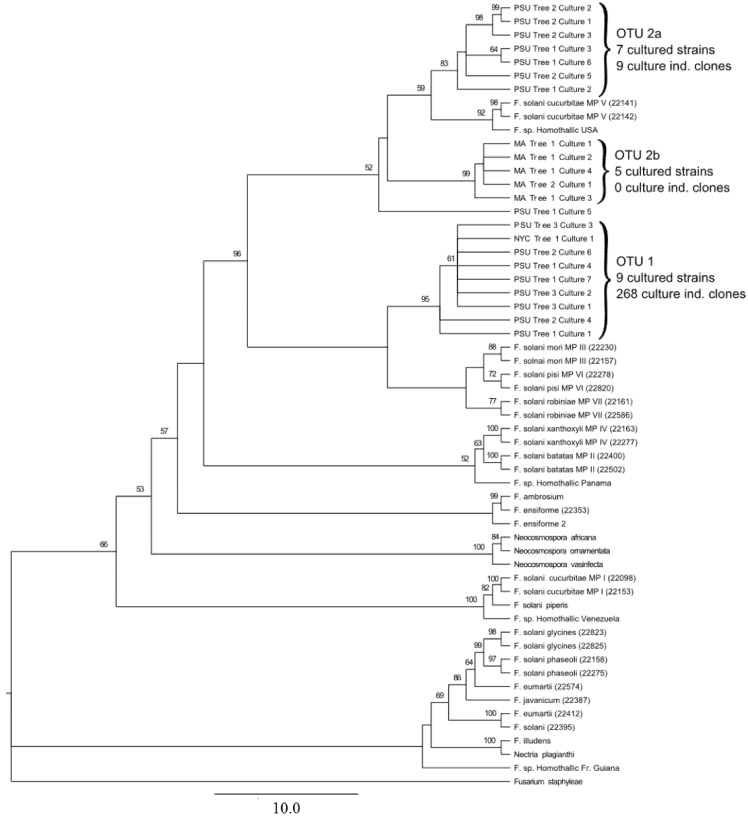
Multilocus phylogenetic analysis of ALB-derived and O’Donnell *Fusarium solani* isolates using the internal transcribed spacer region, the translation elongation factor 1-alpha locus, and the large ribosomal subunit locus. A rooted maximum likelihood tree was generated using a partitioned evolutionary model with sequences from three loci from all isolates cultured from ALB larvae (n = 500 bootstrap replicates). ALB-derived sequences were placed within the *F. solani* species complex, utilizing the dataset of O’Donnell [44]. Mating populations (abbreviated MP) are listed based off of the O’Donnell dataset. Nodes with bootstrap support values > 50 are reported, ML distance scale bar = 10 changes. ALB-derived isolates formed 3 strongly supported monophyletic clades, denoted as OTU1, OTU2a, and OTU2b. A single NYC isolate can be found in OTU1, while all MA-derived isolates were classified into OTU2b. PSU-derived isolates can be observed in both OTU1 and OTU2a.

Multiple locus analysis encompassing the TEF1-α, ITS, and LSU regions from *A. glabripennis*-derived cultures and O'Donnell isolates produced similar tree topology as the TEF1-α single locus analysis, improving bootstrap support on many nodes (Figure 2). One major change that can be noted is that multiple locus analysis now places the outlier sequence (PSU Tree 1 Culture 5) as a neighbor to OTU2 rather than clustering with OTU1. Otherwise, clade 1, which contains all isolates classified as OTU1, and clades 2a and 2b which contain all OTU2-classified isolates, can still be clearly identified. Of particular interest is that the low sequence variability noted within the OTU1 group in the EF-based phylogenetic analysis still persists even when sequence data from two additional loci are integrated, suggesting low genetic diversity among OTU1-derived *F. solan*i isolates. In addition, clade 2b (containing only MA-derived isolates) is still relatively invariant at the nucleotide level in comparison to clade 2a (containing PSU-derived isolates). 

## 3.3. Discussion

In *Anoplophora glabripennis* larvae, collected from 11 trees of six different species at three different geographic locations, we consistently found *F. solani* associated with the insect gut. While the same fungal genotype was not detected in all larval populations analyzed, there was a strong correlation between the *F. solani* strain detected and geographic location. Cultured isolates could be categorized into three clades (Figure 2). Clade 1 contained cultured isolates from PSU- or NYC-populations and culture-independent OTU1. OTU1 was by far the most abundant OTU detected through culture-independent approaches, representing 266/277 (96%) of the clones sequenced and it was detected in every gut sample collected from either NYC-derived or PSU-derived insects (Table 2).

The isolates within OTU1 and clade 1 show very little sequence divergence, suggesting they may be transmitted between generations since isolates from these groups were found not only in colony-derived artificial diet reared insects, but also in their progeny, which were reared in trees in our quarantine facility. Also, the PSU-colony was originally derived from field *A. glabripennis* populations in New York City, where a high abundance of this OTU was also found. It is difficult to determine if OTU1 has a consistent evolutionary relationship with *A. glabripennis* since we were not able to recover it from Worcester, MA-derived insects. Due to difficulty in obtaining samples, we could not perform culture-independent analysis from this geographic location and were limited to performing analysis on cultured isolates. Although OTU2 was significantly less abundant, representing only 4% of clones, it is readily culturable and was often cultured from samples in which OTU2 had not been detected previously in our culture-independent analyses. This suggests that OTU2 may be less abundant in the gut than OTU1, but more amenable to culturing since it represented approximately 40% of our cultured isolates. This could also explain why OTU1 was not detected in MA-derived samples (Table 2 and Table 3). Despite this, OTU1 has low sequence variability and is highly prevalent in our OTU analysis, suggesting a strong relationship with *A. glabripennis*. 

In addition to the highly prevalent *F. solani* OTU1 strain, we also detected a lower prevalence of a second clade of *F. solani* sequences (OTU2, Figure 1 and Figure 2). Unlike OTU1, this OTU was much more heterogeneous than the sequences that comprised OTU2, forming two distinct *A. glabripennis*-derived clades, (clade 2a and clade2b) and was closely related to *F. solani* isolates from mating population V. Clade 2a contains *F. solani* derived from PSU colony-derived culture independent samples from pin oak trees and artificial diet, as well as cultured samples from sugar maple trees. Clade 2b contains all of the sequences from MA-derived insects, and there is low sequence diversity among these samples. The isolates that were most closely related to OTU2 are plant pathogens from mating population V. A similar case is observed for OTU1, as the closest relatives are also plant pathogens (mating populations III, IV and VII). We can speculate that the ancestral source of *F. solani* found in *A. glabripennis* were plant-associated fungal populations, either pathogens or endophytes, that may have been encountered by chance by insects feeding on trees. This could enable these fungi to become associated with the gut either in tandem with OTU1 (as seen in PSU colony-derived insects), or could potentially replace OTU1, which may have occurred in MA-derived insects. The Massachusetts infestation of *A. glabripennis* is thought to be a separate introduction to the U.S. from the New York City populations [1]. This could also explain why MA-derived *F. solani* cultures were distinct from NYC- and PSU colony-derived cultures. 

Regardless of the evolutionary origin of the *F. solani* harbored in the gut of *A. glabripennis*, the consistent association of *F. solani* and sequence homogeneity of *F. solani* isolates within geographic populations of larval *A. glabripennis* suggests that *F. solani* may provide some critical metabolic or biochemical requirement in the gut for larval development. *F. solani* strains are known to have the metabolic ability to degrade cellulose and hemicelluloses, and have some “soft-rot” type lignin degradation abilities [53,54,55,56,57]. Interestingly, members of the *Fusarium solani* species complex are metabolically versatile and can often colonize and thrive on lignocellulose-based substrates, including Kraft and Klason lignin, and produce impressive arrays of laccases, cellulases, xylanses, and enzymes involved in xenobiotic detoxification, suggesting that members of this species complex may harbor efficient lignin-degrading enzymes with potential to help insects overcome many of the challenges associated with feeding in wood [55,58,59,60]. Other potential biochemical capabilities previously detected in *Fusarium* isolates that may benefit insect hosts include sterol synthesis, detoxification of plant-derived allelochemicals and secondary metabolites, and nitrogen scavenging from unconventional substrates, including cyanide and formamide [61,62,63]. The consistent presence of *F. solani* in the insect gut suggests that *A. glabripennis* may be harboring this fungus in order to benefit from its metabolic capabilities. In order to assess the true metabolic and lignin degrading potential of *A. glabripennis F. solani* affiliates, whole genome sequencing of the OTU 1 isolate is currently underway and *in vitro* analyses utilizing model lignin compounds will be conducted to survey for potential lignin degrading enzymes. Furthermore, we are currently investigating the role of *F. solani* in the gut of *A. glabripennis* through metagenomic, transcriptomic and proteomic studies to better understand potential contributions to digestion and gut physiology in *A. glabripennis*.

## 4. Conclusions

Many species in the *F. solani* species complex are notorious plant pathogens, while others are common environmental fungi that are often detected in many diverse habitats [44,64]. The question resulting from this experiment is: Are the *Fusarium* isolates we detected in *A. glabripennis* just environmental contaminants picked up by the beetle during feeding, or are these isolates endosymbionts? It is possible that some of the fungal strains found in a subset of our samples are just environmentally derived or transient fungi that are not associated with this insect. Despite this, a large clade of *F. solani* sequences was detected from all culture independent samples, as well as cultured samples from PSU colony-derived and NYC-derived insect gut fungal samples, which appear to have an intimate association with *A. glabripennis*. Not only did this clade make up the vast majority of the sequences obtained from the clone libraries, but this clade was very distinct from the other *F. solani* strains in our tree or within any previously described mating populations [44]. Also, these strains are not a by-product of colony rearing or geographic distribution, as it was found in both our research colony as well as several collection sites within New York City. To further test this association, analysis of larval guts from Asia, the native range of this insect, would be most informative. In addition, an understanding of the method of transmission of this fungus, and the maintenance of the fungus within the insect gut will be important to confirming the symbiotic nature of the relationship. 
